# A New Algivorous Heterolobosean Amoeba, Euplaesiobystra perlucida sp. nov. (Tetramitia, Discoba), Isolated from Pilot-Scale Cultures of Phaeodactylum tricornutum

**DOI:** 10.1128/spectrum.00817-23

**Published:** 2023-06-28

**Authors:** Hanwen Zhang, Qing He, Xiaoying Jiang, Hongxia Wang, Yulu Wang, Mingyang Ma, Qiang Hu, Yingchun Gong

**Affiliations:** a Institute of Hydrobiology, Chinese Academy of Sciences, Wuhan, China; b State Key Laboratory of Freshwater Ecology and Biotechnology, Institute of Hydrobiology, Chinese Academy of Sciences, Wuhan, China; c College of Advanced Agricultural Sciences, University of Chinese Academy of Sciences, Beijing, China; d Institute for Advanced Study, Shenzhen University, Shenzhen, China; e Shenzhen Institute of Synthetic Biology, Shenzhen Institute of Advanced Technology, Chinese Academy of Sciences, Shenzhen, China; Nanyang Technological University

**Keywords:** algal culture, algivorous protist, amoeba, feeding characterization, Heterolobosea

## Abstract

The diatom Phaeodactylum tricornutum is regarded as a prospective “cell factory” for the high-value products fucoxanthin and eicosapentaenoic acid (EPA). However, contamination with grazing protozoa is a significant barrier to its commercial cultivation. Here, we describe a new species of heterolobosean amoeba, Euplaesiobystra perlucida, which caused the loss of *Phaeodactylum tricornutum* in pilot-scale cultures. Morphological and molecular characteristics distinguish *E. perlucida* from the other species in the genus *Euplaesiobystra*. *E. perlucida* is 1.4 to 3.2 times larger than other *Euplaesiobystra* species in terms of average length/width and maximum length/width of the trophozoites. Unlike Euplaesiobystra salpumilio, *E. perlucida* has no cytostome; *E. perlucida* lacks a flagellate stage, whereas Euplaesiobystra hypersalinica and *E. salpumilio* both display a flagellate stage in their life cycle. The small-subunit rRNA gene sequence of *E. perlucida* shared only 88.02% homology with that of its closest relative, Euplaesiobystra dzianiensis, and had two distinctive regions. Its phylogenetic branch was clustered with one uncultured heterolobosean clone (bootstrap support/posterior probability = 100%/1.00). Results of feeding experiments demonstrated that *E. perlucida* could graze on various unicellular and filamentous eukaryotic microalgae (chlorophytes, chrysophytes, euglenids, and diatoms) and cyanobacteria. *E. perlucida*’s ingestion rate declined exponentially with increasing size of unicellular prey, and *E. perlucida* attained the highest growth rates on *P. tricornutum*. On the basis of its strong ability to graze on microalgae, capacity to form large populations in a short period of time, and capacity to form resistant resting cysts, this contaminant has the potential to cause severe problems in large-scale microalgal culture and merits further attention.

**IMPORTANCE** Heteroloboseans have garnered considerable interest because of their extraordinary ecological, morphological, and physiological diversity. Many heteroloboseans have adapted to various extensive habitats, including halophilic, acidophilic, thermophilic, psychrophilic, and anaerobic habitats. Most heteroloboseans are bacterivores, with a few algivorous species reported. In this study, a new species of algivorous heterolobosean amoeba, *Euplaesiobystra perlucida*, is described as a significant grazer that causes losses in outdoor industrial *Phaeodactylum* cultures. This study provides phenotypic, feeding, and genetic information on a previously unknown heterolobosean, emphasizes the impact of contaminating amoebae in commercial microalgal cultures, and will contribute to the management strategies for predicting this kind of contaminant in large-scale microalgal cultivation.

## INTRODUCTION

The diatom Phaeodactylum tricornutum Bohlin 1897 (1) is widely regarded as a promising “cell factory” for biomanufacturing multiple bioactive substances, such as fucoxanthin, eicosapentaenoic acid (EPA), and chrysolaminarin (2–4). These bioactive substances have antioxidant, antiobesity, antidiabetic, and anticancer properties and can reduce blood cholesterol and protect against cardiovascular and coronary diseases (5, 6). However, commercial production of *P. tricornutum* has not yet been realized due to unstable cultures, low biomass yield, and low bioactive-substance content, which mostly arise as a consequence of protozoan contamination (7, 8). Reports about the protozoan contamination of *P. tricornutum* pilot-scale cultures are much rarer. About 60 years ago, Ansell et al. were the first to find a predator flagellate, *Monas* sp. (Chrysophyceae, Stramenopiles), contaminating such *P. tricornutum* cultures, with the contamination affecting most of their 1,000-L open tanks (9). According to their findings, the appearance and development of *Monas* sp. were frequently linked to the rapid decline of the *P. tricornutum* population (9, 10). In a recent study, seven protozoan strains were identified in the mass culture of *P. tricornutum* in open raceway ponds and tubular photobioreactors, with an undescribed heterolobosean (Heterolobosea, Discoba) amoeba being the most common and destructive predator, resulting in a considerable reduction in biomass and in fucoxanthin and EPA yields (11). However, the amoeba has not yet been precisely described and identified.

The taxon Heterolobosea was established in 1985 by Page and Blanton (12). Currently, this taxon is composed of ~150 species and 35 genera assigned to nine families and two main clades, Pharyngomonada and Tetramitia (13). *Naegleria* is the best known and most studied genus within the Heterolobosea (14). Naegleria fowleri causes primary amoebic meningoencephalitis (15). Naegleria gruberi has been studied mainly as a model organism for amoeba-to-flagellate transformation (16). Both species have been studied in detail for decades. The other heteroloboseans are considerably understudied and undescribed despite their enormous ecological and morphological diversity (14). Most heterolobosean species and genera (~116 species, ~32 genera) have been reported from soils, freshwater and marine habitats, or brackish sediments (13). Other species inhabit a wide range of habitats, including thermal springs (17), hypersaline brines (18), anoxic sediments (19), intestinal tracts of animals (20), and acidic rivers (21). However, reports of heteroloboseans from artificial systems such as microalgal culture systems are remarkably rare. Due to primer bias, the genetic material of these amoebae is not always amplified by the universal small-subunit (SSU) primer, leaving many morphological species without corresponding molecular information (22). In addition, many heteroloboseans have not been successfully cultivated, so morphological and ecological details of these taxa are scarce.

The genus *Euplaesiobystra* is a member of the Tetramitia (Heterolobosea, Discoba) and is well known as a halophile genus (14). Studies of the genus started relatively late. In 2009, Park et al. reported the first comprehensively described species, Euplaesiobystra hypersalinica (the synonym of Plaesiobystra hypersalinica), via morphological and phylogenetic features and established the new genus *Euplaesiobystra* (18). In addition, their observations suggested that *E. hypersalinica* was an “extremely halophilic” eukaryote, with a salinity tolerance of 100 to 300‰ and an ability to grow well at 150 to 200‰ (18). Subsequently, Euplaesiobystra dzianiensis and Euplaesiobystra salpumilio were described, each successively expanding the genus *Euplaesiobystra* (23, 24). Those studies, however, focused mostly on the salinity tolerance of *Euplaesiobystra* amoebae and less on their feeding characteristics. *E. dzianiensis* was the first found to be able to graze on *Arthrospira* filaments, which represented the most abundant cyanobacteria in Lake Dziani Dzaha (Mayotte island, France) (23). More recently, an uncultured *Euplaesiobystra* isolate, CBN AP20, was reported as a contaminant in *Spirulina* production cultures (25). Based on its high density and ability to graze *Spirulina*, isolate CBN AP20 appeared to be the contaminant most harmful to the *Spirulina* in that study (25). Even so, little is known about the feeding characteristics of *Euplaesiobystra* as a predator in microalgal culture systems. In addition, recent Illumina sequencing targeting the V9 region of SSU rDNA revealed six unclassified *Euplaesiobystra* sequences in the Eui-Seong solar saltern, Republic of Korea (26). This finding shows that the taxonomic inventory of *Euplaesiobystra* is far from complete and that the genus is more diverse than previously thought.

In this study, we report a new *Euplaesiobystra* amoeba from pilot-scale cultures of *P. tricornutum* that had a significant impact on the productivity of the culture. Our study focused on the detailed taxonomic description of the new amoeba, including its morphology, life cycle, feeding and digestive processes, and prey preference, together with phylogenetic analysis. Special attention was paid to how the amoeba became a significant contaminant in the *Phaeodactylum* culture, along with consideration of possible management measures to increase the resistance of microalgae to predation or to impair feeding.

## RESULTS

### Impact of the heterolobosean amoeba on mass culture of *P. tricornutum* in open raceway pond outdoor.

**FIG 1:**
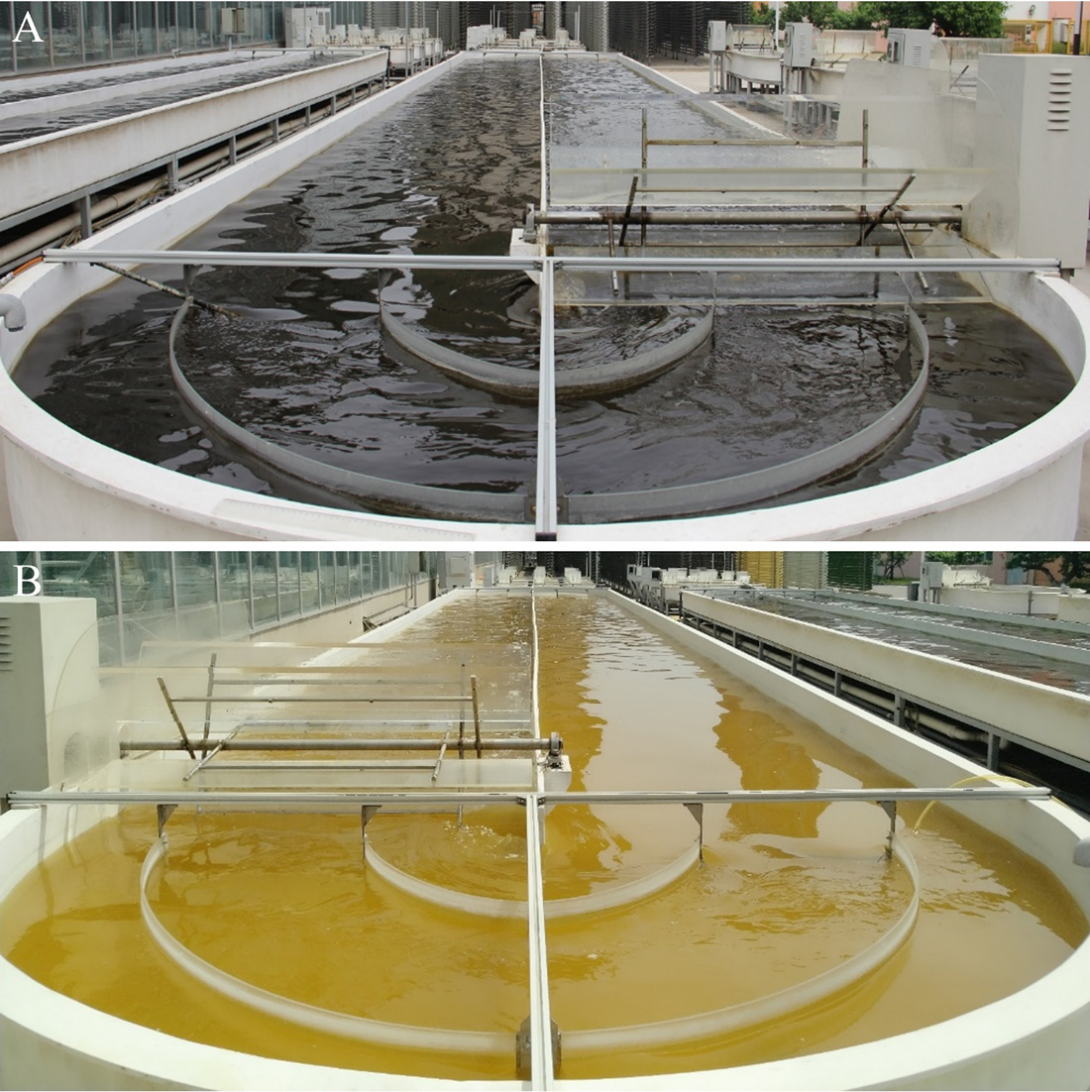
Macroscopic difference between (A) healthy *Phaeodactylum tricornutum* culture and (B) contaminated *P. tricornutum* culture infected with an unknown amoeba (subsequently described as *Euplaesiobystra perlucida* sp. nov.). *P. tricornutum* was cultivated in outdoor 13,000-L open raceway ponds at the R&D facilities of the State Development & Investment Corporation Microalgae Biotechnology Center, Hebei, China (N 39°57′21.97″, E 116°51′35.95″).

**FIG 2:**
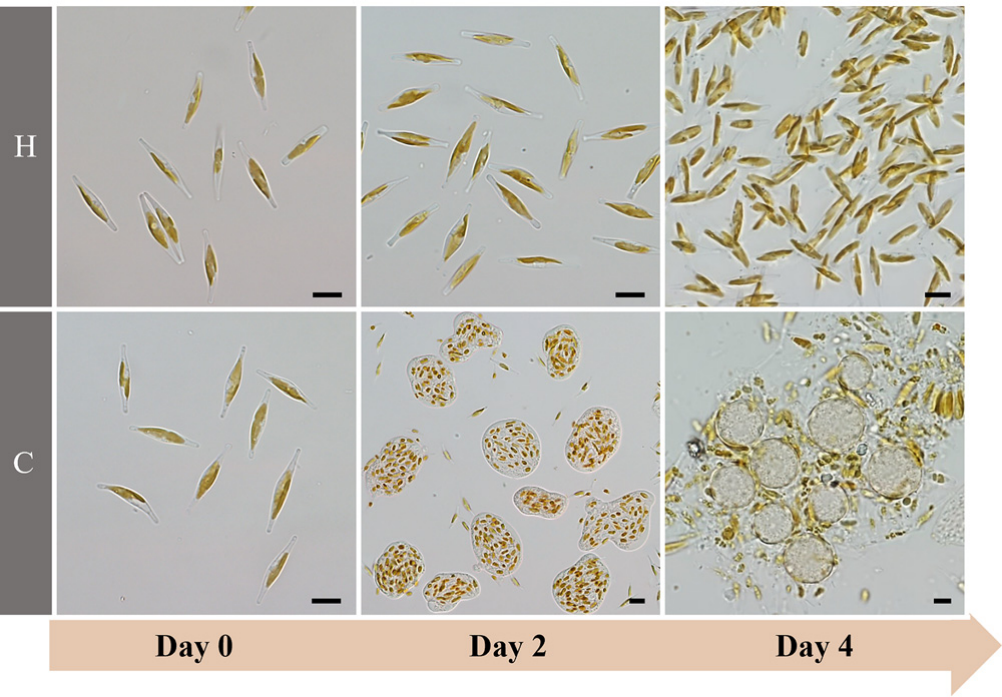
Microphotographs of *P. tricornutum* and an unknown amoeba (subsequently described as *E. perlucida* sp. nov.) in healthy (H) and contaminated (C) *P. tricornutum* culture during cultivation. Bars, 10 μm.

In outdoor 13,000-L open raceway ponds, after 3 to 4 days of cultivation since inoculation, the color of healthy (control) *P. tricornutum* cultures changed from light brown to dark brown (Fig. 1A), whereas the color of contaminated *P. tricornutum* cultures changed from light brown to light yellow (Fig. 1B). Microscopic inspection revealed the presence of an algivorous amoeba in contaminated *P. tricornutum* cultures on the second day after inoculation, and this amoeba grazed extensively on the *P. tricornutum* cells (Fig. 2, “C” panels). On the fourth day of cultivation, the amoeba cells transformed into cysts, forming flocs with the algal cells, and healthy single *P. tricornutum* cells were rarely observed (Fig. 2, “C” panels). In uncontaminated culture, *P. tricornutum* cells grew well and the amoeba was rarely observed (Fig. 2, “H” panels).

**FIG 3:**
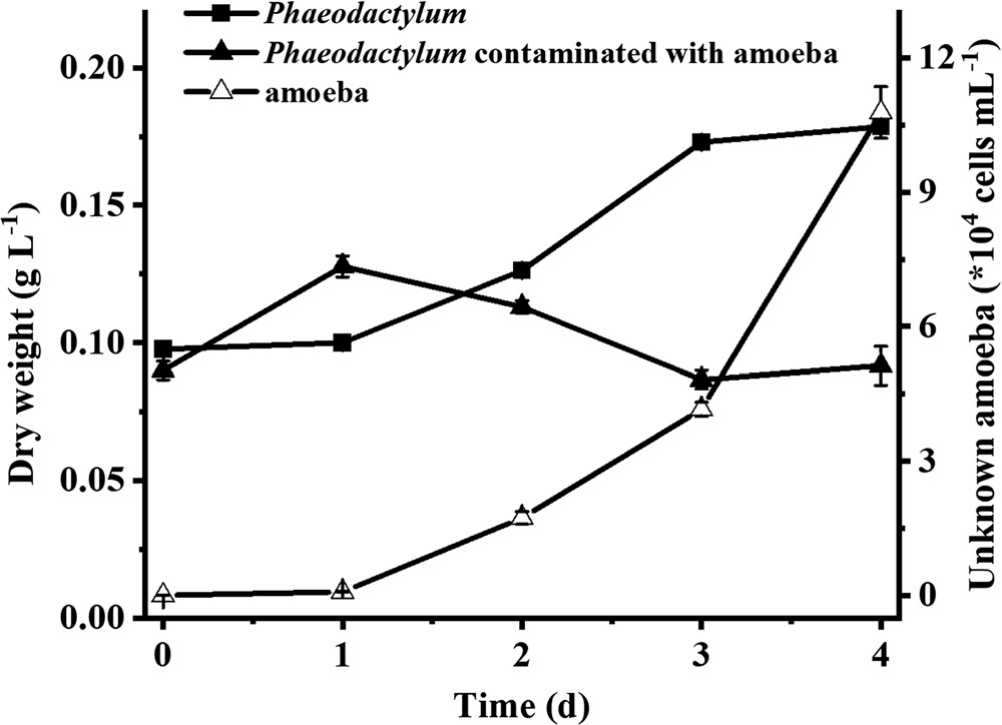
Growth of *P. tricornutum* in outdoor 13,000-L open raceway ponds from 30 April to 4 May 2019. Culture of *P. tricornutum* contaminated with an unknown amoeba (subsequently described as *E. perlucida* sp. nov.) decreased significantly just after a short increase phase (solid triangles), while the numbers of amoeba cells increased over time (open triangles). The uncontaminated control showed lag-, log-, and stationary-phase changes in algal cell numbers (squares). Data are the averages of three counts, and error bars represent standard deviations from the means.

The unknown amoeba had a devastating impact on the growth of *P. tricornutum* (Fig. 3). When the culture was uncontaminated by the amoeba or the amoeba’s concentration was too low to be detected, the dry weight of *P. tricornutum* increased from 0.098 g L^−1^ to 0.18 g L^−1^ within 4 days. In contrast, once the concentration of the amoeba reached approximately 1.0 × 10^4^ cells mL^−1^ in *P. tricornutum* culture, the dry weight of *P. tricornutum* began to decrease significantly, eventually falling below that of the initial inoculation, and the final cell concentration of the amoeba reached values as high as 1.08 × 10^5^ cells mL^−1^. Generally, the predatory amoeba contaminating the *Phaeodactylum* culture occurred from late spring to late autumn (highest temperature, 38°C; lowest temperature, 12°C). According to our observations, the amoeba could occur in any photobioreactors that were exposed to the air or could not be sterilized completely, including pilot-scale cultures located indoors or outdoors (Table 1).

**TABLE 1:** Information on common protozoan contaminants in four *Phaeodactylum* culture systems^*a*^

Culture system	Culture period	Location	Vol (L)	Main contaminants
Tubular photobioreactors	12 Aug–1 Sept 2019	Indoor	520	Unknown amoeba; *Euplotes encysticus*
Open raceway pond	1–10 July 2019	Outdoor	150	Unknown amoeba; *E. encysticus*
Open raceway pond	24 Apr–5 May 2019	Outdoor	1,300	Unknown amoeba; *E. encysticus*
Open raceway pond	10–20 May 2019	Outdoor	13,000	Unknown amoeba; *Chilodontopsis* sp.; *Scuticociliatia* species

a The medium used was F/2.

In addition to the amoeba, some other common protozoans also occurred in the pilot-scale culture systems of *P. tricornutum*, including Euplotes encysticus, *Chilodontopsis* sp., and a species of *Scuticociliatia* (Table 1).

### Morphology and ultrastructure of the amoeba.

**FIG 4:**
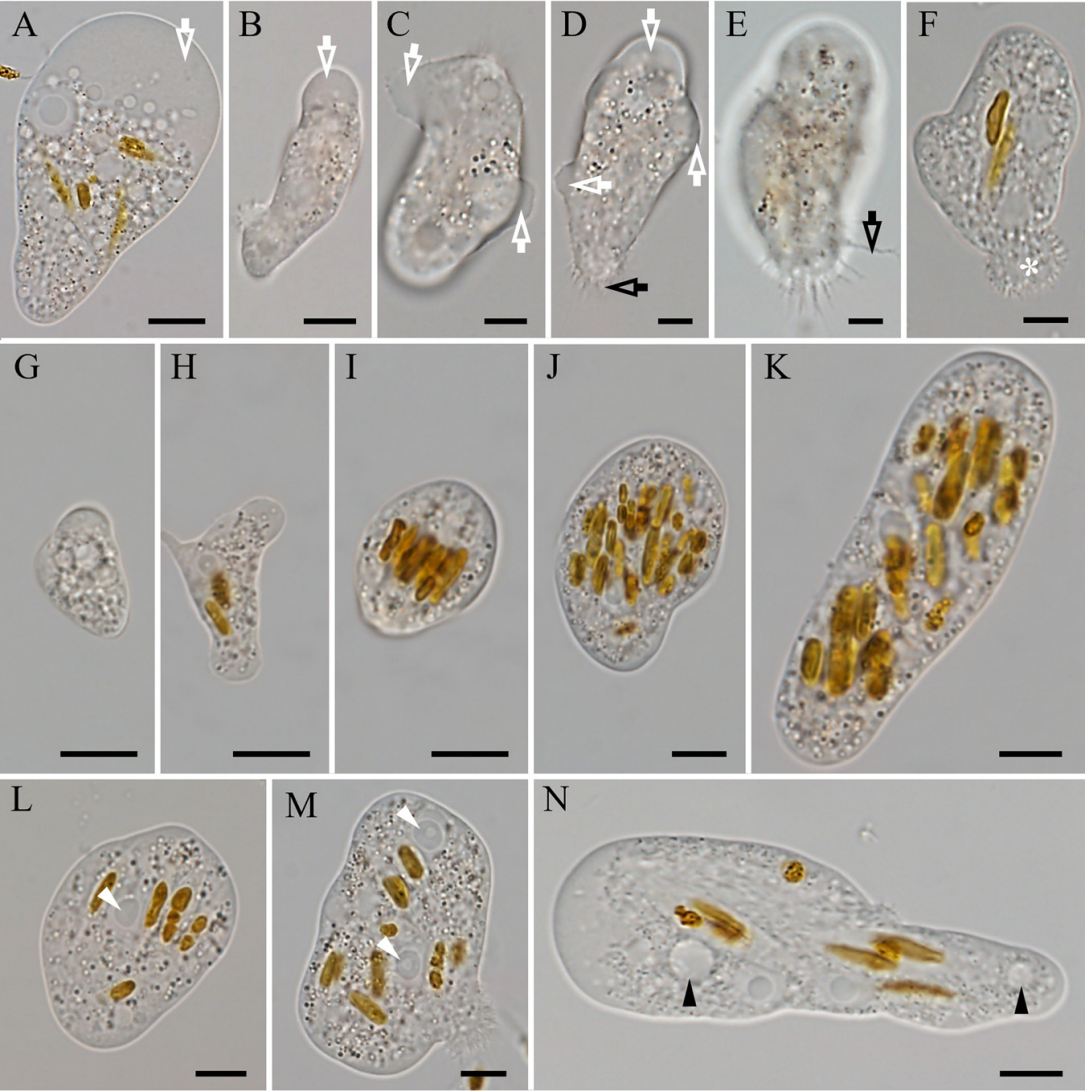
Morphology of trophozoites of *E. perlucida* sp. nov., viewed by differential interference contrast microscopy. (A to F) The various appearances of the trophozoites show the presence of eruptive lobopodia (white arrows; also referred to as hyaloplasmic regions), unbranched uroidal filaments (black arrows), and a caudal bulb (asterisk), with the border between hyaloplasm and granuloplasm sometimes being obvious (A) and at other times not (F). (G to K) Variation in cell size of trophozoites during the feeding process. (L and M) Nuclei (white arrowheads), including an amoeba with one nucleus (L) and one with two nuclei (M). (N) Contractile vacuoles (black arrowheads). Bars, 10 μm.

**(i) Light microscopy.** The trophozoites displayed a cylindrical shape and possessed a marked hyaloplasmic region (also referred to as an anterior hyaline lobopodium), which moved relatively rapidly and was usually solitary but could also occur as two or multiple competing pseudopodia formed by eruptive movement (Fig. 4A to D; also, see Video S1 in the supplemental material). These pseudopodia usually accounted for 10 to 30% of the cell length. Distinct unbranched uroidal filaments were sometimes observed in the trophozoites (Fig. 4D and E). Occasionally, a caudal bulb with fine filaments was present when trophozoites moved (Fig. 4F). Cells were observed to quickly change the direction of movement at a 90° angle. The border between hyaloplasm and granuloplasm in trophozoites was sometimes obvious (Fig. 4A) and sometimes disappeared (Fig. 4F). The trophozoites’ average length and width were 43.5 μm (range, 11.1 to 75.8 μm) and 25.2 μm (range, 8.9 to 44.6 μm), respectively (*n* = 50). The size of the trophozoites varied considerably, depending on the food ingested. As a general rule, the amoeba cell grew as it ingested more food (Fig. 4G to K). The average length/width ratio was 3.8 (range, 1.3 to 5.2) in active cells and 2.3 in slow-moving cells. Trophozoite cells had one to two nuclei (Fig. 4L and M) and one or more contractile vacuoles (Fig. 4N). The flagellate form was not detected at salinities of 3, 15, 30, 45, 75, 100, 150, 200, 250, and 300‰ or at temperatures of 5, 10, 15, 20, 25, 30, 35, 40, 45, and 50°C.

**FIG 5:**
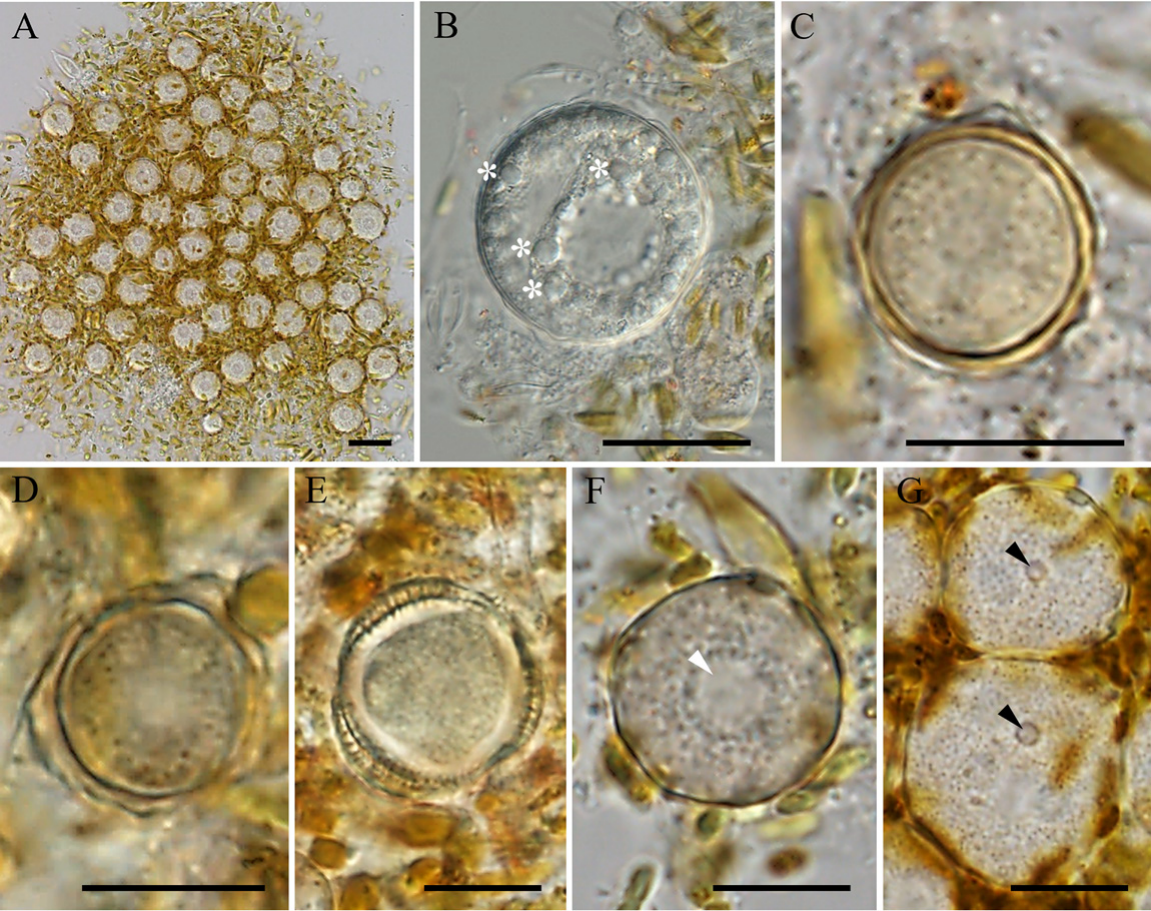
Morphology of cysts of *E. perlucida* sp. nov., viewed by differential interference contrast microscopy. (A) Aggregation of cysts formed from numerous units. (B to E) Possible maturation process of cysts. (B) The cyst is round and has a thin wall and spherical, bead-like structures (asterisks); (C) the mature cyst has a thick double-layered wall; (D) the double-layered wall grows increasingly irregular and the cytoplasm begins to shrink; (E) the cytoplasm has shrunk to an irregular shape, leaving a small gap between it and the cell wall. (F) One central nucleus in a cyst (white arrowhead). (G) Each cyst wall has at least one plugged pore (black arrowheads). Bars, 20 μm (A) and 10 μm (B to G).

The encystment of trophozoites in culture was very synchronous, occurring in less than 24 h once the food algae had been depleted. Cysts could form aggregations of numerous units (Fig. 5A). Young cysts were observed to have a round shape, with a thin wall and spherical, bead-like structures (Fig. 5B). The average diameter of the cysts was 16.6 μm (range, 10.4 to 19.8 μm; *n* = 50). Mature cysts had a thick double-layered wall that became increasingly irregular over time (Fig. 5C and D). Meanwhile, the cytoplasm shrank and took on an irregular shape, leaving a small gap between it and the cell wall (Fig. 5D to F). Most cysts had one central nucleus and at least one plugged pore in the cell wall (Fig. 5G).

**FIG 6:**
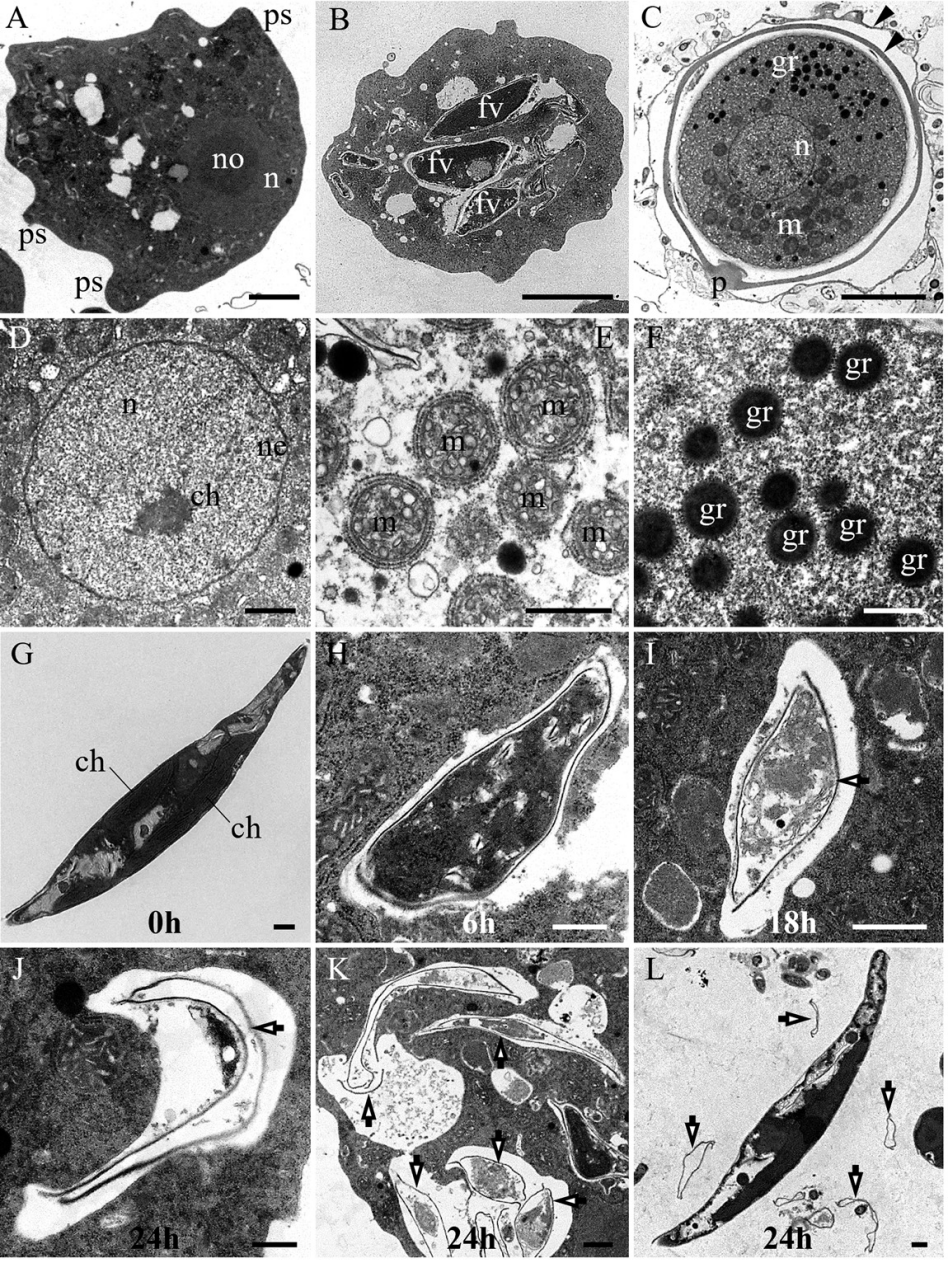
Ultrastructure of *E. perlucida* sp. nov. (A to F) and the process of *P. tricornutum* being digested in amoebae (G to L), viewed by transmission electron microscopy. (A) Section through a trophozoite with electron-dense cytoplasm, with pseudopodia (ps), a nucleus (n), and electron-dense nucleolus (no) visible; (B) well-fed trophozoite cell with some *Phaeodactylum* cells in separate food vacuoles (fv); (C) cyst exhibiting a two-layered wall (arrowheads), a central nucleus (n), a single cyst pore (p), clusters of mitochondria (m), and clusters of electron-dense granules (gr); (D) details of a nucleus (n), displaying a visible nuclear envelope (ne) and strands of condensed chromatin (ch); (E) details of mitochondria (m), which are surrounded by rough endoplasmic reticulum and display discoidal cristae; (F) high-magnification view of electron-dense granules (gr); (G) uningested *Phaeodactylum* cell with intact structures such as the chloroplast (ch); (H) *Phaeodactylum* cell in the early digested phase, displaying comparatively intact and deformed morphology; (I) *Phaeodactylum* cell in the middle digested phase, with partially hydrolyzed cytoplasm and undigested cell walls (arrow); (J and K) *Phaeodactylum* cell in the late digested phase, showing only unhydrolyzed cell walls (arrows); (L) undigested cell walls of *Phaeodactylum* expelled from the amoebae (arrows). Bars, 5 μm (A to C), 1 μm (D, E, G, I, K, and L), and 500 nm (F, H, and J).

**(ii) Ultrastructure.** In sections of the trophozoites, the electron-dense cytoplasm, the blunt round pseudopodia, a distinct nucleus, and an electron-dense nucleolus were observed (Fig. 6A). The nucleus was located close to the cell membrane (Fig. 6A). In well-fed trophozoites, *Phaeodactylum* cells were frequently seen in separate food vacuoles (Fig. 6B). Cysts were spherical. The number of layers in the cyst wall depended on the stage of cyst development, with mature cysts having two-layered walls (Fig. 6C). Cysts exhibited a central nucleus, a cyst pore, clusters of mitochondria, and clusters of electron-dense granules (Fig. 6C). A nuclear envelope and strands of condensed chromatin were evident in the nucleus (Fig. 6D). The mitochondria were surrounded by rough endoplasmic reticulum and possessed discoidal cristae (Fig. 6E). Electron-dense granules were spherical and had distinct boundaries (Fig. 6F).

The process of algal digestion in amoebae was studied using transmission electron microscopy. In general, uningested *Phaeodactylum* cells were found to have complete morphology and structure, with chloroplasts visible (Fig. 6G). In the early stage of digestion, after about 6 h, *Phaeodactylum* cells were deformed but relatively intact (Fig. 6H). After about 18 h, most of the cytoplasm of *Phaeodactylum* cells was hydrolyzed, but undigested cell walls remained (Fig. 6I). In the late stage of digestion, after about 24 h, the cytoplasm of *Phaeodactylum* cells had been fully hydrolyzed, but the cell walls still remained relatively intact (Fig. 6J and K). Undigested algal cell walls expelled by the amoeba were detected throughout the sample sections (Fig. 6J and K).

### Molecular phylogeny of the amoeba.

The SSU rRNA gene sequence amplified from the unknown amoeba was 1,831 bp long. The two sequences most similar to this amoeba, as revealed by a BLAST search of the GenBank database, were a partial SSU sequence from an uncultured heterolobosean isolate, CBN AP20 (accession no. MF490458; 1,121 bp, 97.91% identity), and *Euplaesiobystra dzianiensis* (accession no. MN969059, deposited as Heterolobosea sp. VDe-2020a isolate DD2; 88.02% identity). A predicted helix 17_1 in the secondary structure of the SSU rRNA was present in the amoeba (Fig. S2). Our sequence, together with 60 previously published Tetramitia sequences and two sequences of Pharyngomonada as an outgroup (total of 63 sequences; >1,000 bp; 1,689 analyzed sites), was used to conduct comparative analyses.

**FIG 7:**
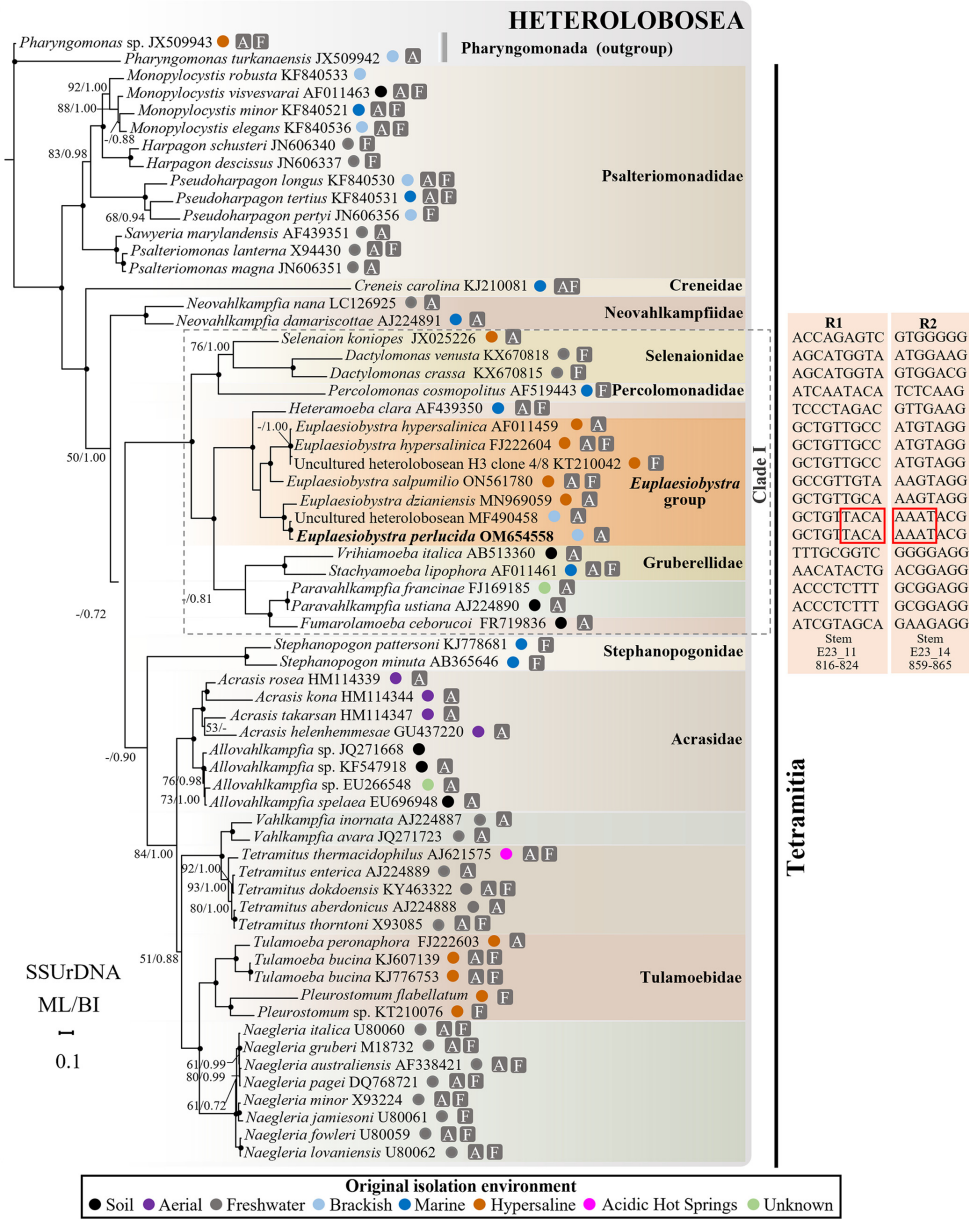
ML phylogeny of Tetramitia (with Pharyngomonada as an outgroup) based on comparisons of SSU rRNA gene sequences (63 sequences, 1,689 analyzed sites; GTR+Γ+I model), showing the position of *E. perlucida* sp. nov. Bootstrap support values (ML analysis) and posterior probabilities (BI) are shown as ML/BI. Support values of <50% and posterior probabilities of <0.7 are not shown or are indicated by dashes. The black circles represent support values at or above 95%/0.95. The environmental origin for sequences is indicated by colored circles. A, species with an amoeba phase; F, species with a flagellate phase, when available. In the sequences, the areas delineated by the red boxes are the distinctive regions of *E. perlucida* that are consistent with one uncultured heterolobosean and distinct from other families/genera/species in clade I and correspond to the phylogenetic grouping. Two distinctive regions are located at helix E23_11 (nucleotides 816 to 824) and helix E23_14 (nucleotides 859 to 865). The nucleotide positions were based on the SSU rRNA gene sequence of Selenaion koniopes (JX025226). The scale bar represents 0.1 nucleotide substitution per site.

Two distinctive regions were revealed in the sequence of the new heterolobosean amoeba. The first was nucleotides 816 to 824 in helix E23_11, and the second was nucleotides 859 to 865 in helix E23_14 (Fig. 7). The topologies generated by maximum-likelihood (ML) and Bayesian inference (BI) analyses were nearly identical, with the final result presented here being the ML topology (Fig. 7). According to phylogenetic analyses based on the SSU rRNA gene, our amoeba, a brackish-water species, belonged to the genus *Euplaesiobystra* and was closest to one environmental brackish-water sequence (MF490458; 1,121 bp; bootstrap support ML, 100%; posterior probability, 1.00). Both the latter sequence and our amoeba were part of the fully supported clade (unclassified clade in Tetramitia) that included *E. dzianiensis* (MN969059), *Euplaesiobystra hypersalinica* (FJ222604), *Plaesiobystra hypersalinica* (AF011459), and *Euplaesiobystra* sp. (KT210042). *Heteramoeba* was the sister group nearest to *Euplaesiobystra*.

### Ability of the amoeba to graze on other microalgae and cyanobacteria.

The 32 microalga/cyanobacterium strains tested as the prey of the amoeba were of various sizes, shapes, and motilities (Table 2). Microscopic observation revealed that the amoeba could graze on the majority of the microalga strains and all the cyanobacteria (Table 2), showing a broad feeding spectrum of prey and algal cultures that it may potentially harm. Based on the ability of the amoeba to feed on the prey, the 32 microalga/cyanobacterium strains were separated into three groups.

**TABLE 2:** Potential capacity of *E. perlucida* to prey upon various commercial microalgae

Algal group	Species	Strain	Morphology	Mobile	Amoeba category^*a*^
Chlorophytes	*Chlorella sorokiniana*	CMBB146	Unicellular	No	++
	*Chlorella vulgaris*	FACHB-8	Unicellular	No	++
	*Chlorella pyrenoidosa*	FACHB-9	Unicellular	No	++
	*Chlorella sorokiniana* ^*b*^	CGMCC11801	Unicellular	No	++
	*Chlorella sorokiniana*	FACHB-275	Unicellular	No	++
	*Scenedesmus acuminatus*	CMBB220	Unicellular	No	−
	*Scenedesmus acuminatus*	CMBB102	Unicellular	No	−
	*Haematococcus pluvialis*	CCAP34/7	Unicellular	Yes	−
	*Haematococcus pluvialis*	CMBB361	Unicellular	Yes	−
	*Nannochloropsis oceanica* ^*b*^	IMET1	Unicellular	No	++
	*Dunaliella salina*	CMBB318	Unicellular	Yes	++
	Chlamydomonas reinhardtii	CC124	Unicellular	Yes	+
	*Ulothrix* sp.	FACHB-1747	Filamentous	No	−

Chrysophytes	*Isochrysis zhanjiangensis*	CMBB282	Unicellular	Yes	++
	*Isochrysis* sp.	CMBB112	Unicellular	Yes	++
	*Poterioochromonas malhamensis*	CGMCC11620	Unicellular	Yes	+
	*Poterioochromonas malhamensis*	CCMP1862	Unicellular	Yes	+

Euglenids	Euglena gracilis	FACHB-848	Unicellular	Yes	+
	Euglena gracilis var. bacillaris	FACHB-850	Unicellular	Yes	+
	*Euglena* sp.	FACHB-1914	Unicellular	Yes	+

Diatoms	*Phaeodactylum tricornutum* ^*b*^	UTEX640	Unicellular	No	++
	*Chaetoceros* sp.^*b*^	HR-CH301	Unicellular	No	++
	*Navicula* sp.	FACHB-1996	Unicellular	No	++
	*Nitzschia* sp.	FACHB-1054	Unicellular	No	++

Cyanobacteria	Microcystis aeruginosa	FACHB-928	Unicellular	No	++
	Microcystis aeruginosa ^*b*^	FACHB-905	Unicellular	No	++
	*Microcystis flos-aquae* ^*b*^	FACHB-1028	Unicellular	No	++
	Microcystis aeruginosa	FACHB-942	Unicellular	No	++
	*Arthrospira platensis*	FACHB-SP2A	Filamentous	No	+
	*Synechocystis* sp.^*b*^	FACHB-898	Unicellular	No	++
	*Desertifilum salkalinema*	FACHB-7200	Filamentous	No	++
	*Synechococcus* sp.^*b*^	FACHB-805	Unicellular	No	++

a Results were used to categorize amoebae as “most suitable/rapid growth (++),” “suitable/limited growth (+),” and “not suitable/no growth (−).”

b Organism used in quantitative experiments.

**FIG 8:**
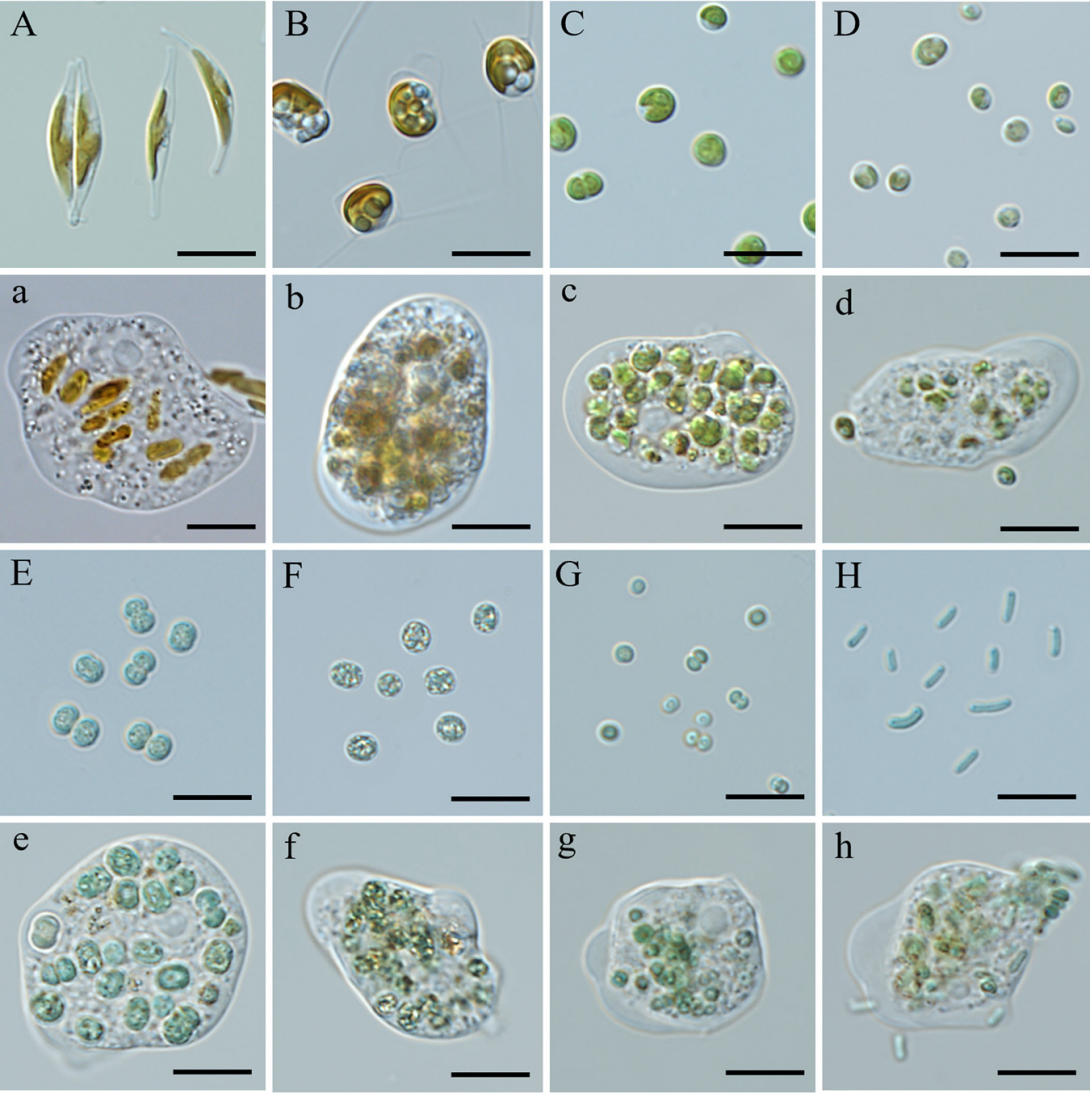
Cell morphology of eight microalgal/cyanobacterial strains (A to H) and feeding behavior of *E. perlucida* sp. nov. on these strains (a to h), viewed by light microscopy. (A and a) *Phaeodactylum tricornutum* (UTEX 640); (B and b) *Chaetoceros* sp. (HR-CH301); (C and c) *Chlorella sorokiniana* (CGMCC11801); (D and d) *Nannochloropsis oceanica* (IMET1); (E and e) Microcystis aeruginosa (FACHB-905); (F and f) *Microcystis flos-aquae* (FACHB-1028); (G and g) *Synechocystis* sp. (FACHB-898); (H and h) *Synechococcus* sp. (FACHB-805). Bars, 10 μm.

**FIG 9:**
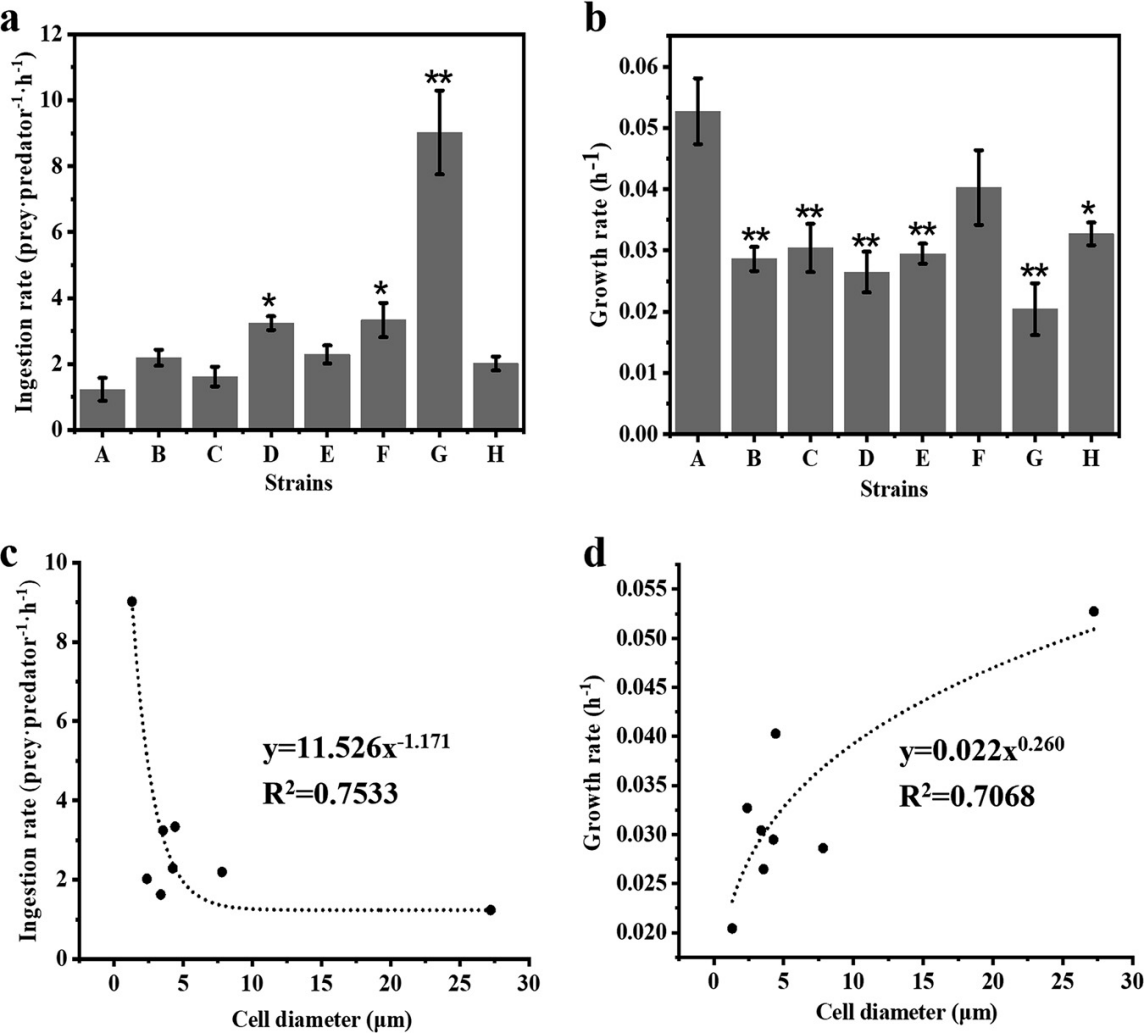
Ingestion rate (a) and growth rate (b) of *E. perlucida* sp. nov. on eight microalgal/cyanobacterial strains and relationship between microalgal/cyanobacterial cell diameter and the amoeba’s ingestion (c) or growth (d) rate. The ingestion rate was used to evaluate the reduction in algal cells by grazing per amoeba per hour, and growth rate represents the exponential increase at a constant rate throughout time interval. Bars: A, *Phaeodactylum tricornutum* (UTEX640); B, *Chaetoceros* sp. (HR-CH301); C, *Chlorella sorokiniana* (CGMCC11801); D, *Nannochloropsis oceanica* (IMET1); E, Microcystis aeruginosa (FACHB-905); F, *Microcystis flos-aquae* (FACHB-1028); G, *Synechocystis* sp. (FACHB-898); H, *Synechococcus* sp. (FACHB-805). *, 0.01 < *P* ≤ 0.05 (significant difference); **, *P* ≤ 0.01 (extremely significant difference).

One group of strains, described as “most suitable/rapid growth (++),” served as food organisms and supported rapid growth of the amoeba population. In cocultures with these strains, the amoeba depleted microalgal/cyanobacterial cells within 3 days. This group contained 20 microalgal/cyanobacterial strains, including seven chlorophytes (*Chlorella*, *Nannochloropsis*, and *Dunaliella*), two chrysophytes (*Isochrysis*), four diatoms (*Phaeodactylum*, *Chaetoceros*, *Navicula*, and *Nitzschia*), and seven cyanobacteria (*Microcystis*, *Synechocystis*, *Desertifilum*, and *Synechococcus*). Eight nonmotile, unicellular microalgal strains (Table 2) were chosen from this group in order to investigate the connection between the amoeba’s feeding and growth rates and the size of the prey cell. The eight strains varied in size (Fig. 8A to H). Microscopic results showed that there were numerous microalgal/cyanobacterial cells inside the amoeba cells during the experiments (Fig. 8a to h). The amoeba’s ingestion rate on these eight microalgal/cyanobacterial species varied greatly, ranging from 9.02 to 1.23 prey predator^−1^ h^−1^, with the highest ingestion rate being on *Synechocystis*, followed by Microcystis flos-aquae and Nannochloropsis oceanica (Fig. 9a). The ingestion rate declined exponentially with an increase in prey size (Fig. 9c) and was lowest when the prey was *Phaeodactylum* with a size of 27.23 μm. The growth rates of the amoeba on these eight microalgal/cyanobacterial species ranged from 0.053 to 0.020 h^−1^, with the highest growth rate being on *Phaeodactylum*, followed by *M. flos-aquae* and *Synechococcus* (Fig. 9b). There were significant (0.01 < *P* ≤ 0.05) or extremely significant (*P* ≤ 0.01) differences between the growth rate achieved with *Phaeodactylum* as the prey and that achieved when the other microalgal/cyanobacterial species were the prey, except for *M. flos-aquae* (Fig. 9b). The growth rate of the amoeba increased exponentially with an increase in prey size (Fig. 9d).

The second group of strains, described as “suitable/limited growth (+),” also served as food organisms but were less well able to support the growth of the amoeba population. In this group, the amoeba reduced the prey population to nearly zero by the end of the 7-day experiment. This group contained seven different strains, including one chlorophyte (*Chlamydomonas*), two chrysophytes (*Poterioochromonas*), three euglenids (*Euglena*), and one cyanobacterium (*Arthrospira*).

The third group of algae, regarded as “not suitable/no growth (−),” were not used as food organisms by the amoeba, and the amoeba showed no signs of interaction with this group. This group included only five chlorophytes (*Scenedesmus*, *Haematococcus*, and *Ulothrix*) (Table 2). With these algae as food, the amoeba began to form cysts within hours during the experiment.

## TAXONOMY

Euplaesiobystra perlucida sp. nov. (Heterolobosea, Page & Blanton, 1985; Tetramitia, Cavalier-Smith 1993; *Euplaesiobystra*, Park et al. 2009). *per- lūcidus*, *- a*, *- um* [Latin] = transparent. The epithet *perlucida* refers to the trophozoites being transparent.

Trophozoites: cylindrical; 43.5 μm (range, 11.1 to 75.8 μm) and 25.2 μm (range, 8.9 to 44.6 μm) in average length and width, respectively; average length/width ratio is 3.8 and 2.3 in active cells and slow-moving cells, respectively; size of trophozoites varies considerably with ingestion; eruptive movement; usually solitary; sometimes with uroidal filaments and a caudal bulb during motion; no cytostome; one or two nuclei. Flagellates were not detected in various salinity and temperature treatments. Cysts: 16.6 μm (range, 10.4 to 19.8 μm) average diameter; young cysts round and with a thin wall and spherical, bead-like structures; mature cysts round or irregular in shape and with a thick double-layered wall; one central nucleus and at least one plugged pore.

Differs from *E. dzianiensis* in length and width (11.1 to 75.8 μm versus 17 to 33 μm, 8.9 to 44.6 μm versus 8 to 20 μm). Differs from *E. salpumilio* in length and width (11.1 to 75.8 μm versus 11.5 to 23.2 μm, 8.9 to 44.6 μm versus 5.1 to 14.5 μm) and in the absence of cytostome and flagellate stage. Differs from *E. hypersalinica* in length and width (11.1 to 75.8 μm versus 19 to 41 μm, 8.9 to 44.6 μm versus 9 to 16 μm) and in the absence of flagellate stage. Differs from three known species of the genus *Euplaesiobystra* in the feeding spectrum (eukaryotic microalgae and cyanobacteria versus prokaryotes).

A permanent preparation (one resin block for TEM), which serves as a name-bearing hapantotype (article 73.3, ICZN), has been deposited in the Protists Collection at the Department of Zoology of the Natural History Museum (London, UK) with registration number NHMUK 2023.2.9.1. Deposited in the Freshwater Algae Culture Collection at the Institute of Hydrobiology, Wuhan, China, with registration number FACHB-3575 at http://algae.ihb.ac.cn/English/. Cells of the hapantotype are shown in Fig. 4 and Fig. 5. The sequence of the type-generating strain (SSU rRNA gene) has accession number OM654558. The ZooBank Life Science identifier (LSID) is urn:lsid:zoobank.org:pub: 8FE9E9FE-43F2-470C-B666-4E47BDDB070A.

Habitat and locality are brackish water (20 ppt) and outdoor 13,000-L raceway ponds in the State Development & Investment Corporation (SDIC) Microalgae Biotechnology Center, Hebei, China.

## DISCUSSION

### Rationale for the introduction of a new species in the phylum Heterolobosea.

In this study, a new species in the phylum Heterolobosea is described. It has obvious unbranched uroidal filaments and a caudal bulb in the amoeba stage (Fig. 4), at least one plugged pore on the cyst (Fig. 5G and 6C), and a putative secondary structure of the helix 17_1 in the SSU rRNA gene (Fig. S2) and feeds by engulfment. All of the aforementioned characteristics are typical of the phylum Heterolobosea (13, 14). Moreover, this amoeba was grouped with known members of the genus *Euplaesiobystra* in the phylogenetic tree (Fig. 7). We therefore concluded that this species belongs to the Heterolobosea, and we assigned it to the genus *Euplaesiobystra* with the name Euplaesiobystra perlucida.

According to previous studies, the genus *Euplaesiobystra*, which was established in 2009 (23), contains three well-described species, namely, *E. dzianiensis* (18), *E. hypersalinica* (23), and *E. salpumilio* (24). *E. perlucida* shows morphological and autecological characteristics that are distinct from those of these three species. Regarding average length/width and maximum length/width in the trophozoite stage, *E. perlucida* appears to be 1.4 to 3.2 times larger than the three existing species of *Euplaesiobystra* (23, 24). The average and maximum length/width of *E. perlucida* were 43.5/25.2 μm and 75.8/44.6 μm, respectively; for *E. dzianiensis*, they are 22/13 μm and 33/20 μm (23); for *E. salpumilio*, they are 17.6/9.2 μm and 23.2/14.5 μm; and for *E. hypersalinica*, they are 30.5/12.0 μm and 41/16 μm (Table S1) (24). Unlike *E. salpumilio* (24), *E. perlucida* has no clear cytostome. Regarding life cycle, *E. perlucida* lacks a flagellate stage, whereas *E. hypersalinica* (23) and *E. salpumilio* (24) display a flagellate stage in their life cycle (Table S1) (18, 24). Regarding feeding characteristics, *E. perlucida* displayed a broad spectrum of cyanobacterial and eukaryotic microalgal prey, whereas the other three species have been documented to feed only on prokaryotes/bacteria (Table S1) (18, 23, 24).

According to our molecular phylogenetic results, the new heterolobosean in our study did not group with any known species (Fig. 7) but clustered with one uncultured heterolobosean detected in a brackish environment (25), with which it shared two different sequence regions (Fig. 7). Given its morphological and feeding differences as well as a genetic divergence from the other recorded species in the genus *Euplaesiobystra*, we describe *E. perlucida* as a new species. This discovery of a new species, together with the report of the six unclassified *Euplaesiobystra* sequences from the Republic of Korea (26), supports the view that *Euplaesiobystra* is more diverse than previously realized and that the taxonomic inventory of *Euplaesiobystra* is still far from complete. Continuous investigation of *Euplaesiobystra* is therefore needed to better understand its phenotypic, ecological, and genetic diversity.

### Impact of *E. perlucida* on pilot-scale cultures of *P. tricornutum* in outdoor open raceway ponds.

There are a few systematic studies of algivorous amoebae that contaminate commercial microalgal/cyanobacterial cultivation systems, and several important culprits have been identified. Investigations at Arizona State University revealed that the vampyrellid Vernalophrys algivore caused a decline in the quality and productivity of Scenedesmus dimorphus cultures cultivated in raceway ponds and photobioreactors, with contamination by the vampyrellid occurring at any time of the year (27). Zhang et al. reported another vampyrellid, Kinopus chlorellivorus, that resulted in the etiolation, flocculation, and collapse of Chlorella sorokiniana cultures grown in raceway ponds (28). The impact of amoebae is not, however, restricted to freshwater microalgal taxa. In Dunaliella salina ponds, the amoeba *Naegleria* sp. and a ciliate (Euplotes persalinus) were found to rapidly decimate the algal culture when the salinity dropped below 20% (wt/vol) NaCl (29). Outdoor mass cultures of *Arthrospira* sp. in the north of Chile were contaminated with an amoeba (*Amoeba* sp.) and a rotifer (*Brachionus* sp.), which generated cellular breakdown and the eventual death of the culture. According to the amoeba contaminations that were described, if an invasion was not controlled quickly, cultures would perish within days (30). Spirulina platensis cultures grown at an industrial scale of 500 to 2,000 m^2^ in Brazil occasionally became suddenly contaminated with unknown amoebae (31). If the invasion was not dealt with early enough, the amoebae could destroy the culture within 3 days (31, 32). All these studies suggest that algivorous amoebae play a critical role in the demise of microalgal mass cultures. In the context of ensuring productivity, grazing is a widespread problem and there is a growing volume of literature on the topic (33).

In this study, we report a heterolobosean amoeba, *E. perlucida*, causing significant damage to *P. tricornutum* culture in pilot-scale production. This predatory amoeba contaminating the *Phaeodactylum* culture was prevalent in diverse culture systems (Table 1). Our prior research showed that when *P. tricornutum* culture in a 1,300-L open raceway pond was significantly contaminated by this amoeba, the biomass productivity, cellular fucoxanthin concentration, and EPA concentration dropped by 84.62%, 96.80%, and 88.39%, respectively (11).

There are several possible reasons why *E. perlucida* causes catastrophic losses in *P. tricornutum* culture systems. The first is the strong grazing capability and high cell concentration of *E. perlucida*. *E. perlucida* was capable of moving and engulfing prey food using its pseudopodia, and one amoeba can engulf as many as 60 *P. tricornutum* cells within tens of minutes (11). The amoeba’s cell size is quite malleable and can increase by 20 to 30 times throughout the feeding process (11). According to the results of this study and earlier studies, seven protozoan species have been observed to occur in *P. tricornutum* cultures, but only *E. perlucida* and the ciliate Euplotes encysticus have been found to graze on *P. tricornutum* cells, and microscopic examination reveals that the grazing ability of *E. perlucida* is stronger than that of *E. encysticus*. Furthermore, *E. perlucida* reproduces rapidly under conditions of adequate food. It can increase in abundance by a factor of 2 to 6 in just 24 h (11), and in the present study, the concentration of amoebae in pilot-scale cultures reached values as high as 1.08 × 10^5^ cells mL^−1^ (Fig. 3). In conclusion, *E. perlucida* can quickly consume a large number of algal cells after contaminating culture systems and has the capacity to achieve a high concentration.

The second reason is the capacity of *E. perlucida* to form cysts in adverse environments. Based on previous research, *Euplaesiobystra* can encyst, and these cysts remain viable for at least 60 days at 30‰ salinity (18). This indicates that the cysts may survive in *P. tricornutum* culture (20 to 30‰) for a long time. Microalgal cultivation is a step-by-step, scale-up process in which the culture in the last step serves as the seed culture for the next cultivation (34, 35). Therefore, once the seed culture is contaminated with cysts, the subsequent cultures are more susceptible to contamination and prone to collapse more quickly. Moreover, it is difficult to completely remove the cysts when cleaning photobioreactors, and this can easily induce a new round of infection. Contamination from the air may also occur; Cho suggested that the cysts of halophilic protozoa may be transported by aerosols (36). For cultures in open raceway ponds, there is a higher risk of becoming contaminated than in more closed systems, as many *E. perlucida* cells can enter the culture system at any time from the air as well as from the seed culture. We propose that this is the main reason why the contamination caused by the heterolobosean amoeba (the same species as *E. perlucida*) in our open pond systems was particularly pronounced.

### Feeding characteristics of *Euplaesiobystra* amoebae.

Previous studies on the autecology of the genus *Euplaesiobystra* have been devoted to salinity tolerance (18, 23, 24), with only a few, scant investigations on its feeding habits. Currently known described species in the genus *Euplaesiobystra* have the following feeding characteristics: *E. hypersalinica* is a bacterivore (18); *E. salpumilio* feeds on prokaryotes that have been grown on LB (Luria-Bertani) culture medium (15); *E. dzianiensis* grazes on the filamentous cyanobacterium Arthrospira fusiformis, and it has been suggested that this amoeba’s distribution pattern follows that of *Arthrospira* in a thalassohaline lake (23). In this study, our amoeba, *E. perlucida*, fed on 27 strains of microalgae/cyanobacteria through phagocytosis, exhibiting a broad feeding range and rapid growth on both eukaryotic and prokaryotic taxa (Table 2). Our results complement the known feeding characteristics of *Euplaesiobystra* and suggest that *Euplaesiobystra* may display more trophic complexity than previously thought.

Our amoebae exhibited different ingestion and growth rates on various-sized unicellular microalgae/cyanobacteria (Fig. 9). The ingestion rate of *E. perlucida* declined exponentially (Fig. 9c) and its growth rate increased exponentially (Fig. 9d) with increasing prey size. Many factors, including size, shape, swimming behavior, biochemical composition, surface structure variation, and physiology of the prey, have been reported to influence the feeding characteristics of protozoa on prey (37). Although the effect of shape, biochemical composition, and other factors on the ingestion rate of *E. perlucida* on different microalgae/cyanobacteria in our study could not be excluded completely, prey size has always been thought of as the first-order determinant in other studies (38). Dillon and Parry reported that the amoebae *Echinamoeba* sp. and *Acanthamoeba* sp. could ingest all synechococci presented to them but showed higher ingestion rates with the smaller *Synechococcus* strains than the larger strains (39). An inverse relationship between picocyanobacterial cell size and ingestion rate has also been recorded for heterotrophic nanoflagellates and ciliates (40). In addition, the grazing rate of Poterioochromonas malhamensis was found to decline exponentially as algal size increased, reaching an extremely low value when the prey size reached >8 μm (41). Reports of prey size versus predator growth rate, however, are relatively rare for amoebae, although they are available for other protozoan taxa. Phagotrophic dinoflagellates, for example, can grow at predator/prey size ratios between 0.15:l and 5.2:l but show optimal growth on prey approximately as large as themselves (42). Generally, the total nutritional value of microalgae/cyanobacteria increases with cell size. If the prey has high nutritional value, the predator’s growth rate will increase and its ingestion rate will decrease (43). The mechanism of this phenomenon may be explained by the compensatory feeding response, where high ingestion on smaller prey compensates for lower nutritional quality to satisfy elemental content demand (44).

Studies on feeding can provide insights into possible management approaches that may be employed to reduce the impact of predators on microalgal cultures. For specific predators with a single feeding strategy, feeding selectivity is more of a function of prey properties, so changing prey properties can reduce the predator’s feeding (45). Strom et al. (46) found that the deletion of the SwmA gene, which is involved with the synthesis of the S-layer in the cell wall of *Synechococcus* sp. strain WH8102, could enable this organism to resist predation by flagellates and ciliates. In addition, the presence of the giant SwmB protein in the cell surface of *Synechococcus* sp. strain WH8102 may confer resistance against Oxyrrhis marina predation (47). The majority of these resistance verification experiments were carried out in the laboratory, so whether these algal strains remain resistant to predation in outdoor environments needs to be further explored. Even so, this approach, whereby the properties of the alga are altered to increase its resistance to predators, is still considered the best solution for avoiding contamination of microalgal cultures, given that resistant strains are inexpensive, safe, and pollution free (33). With the commercialization of the biomass of *P. tricornutum* and its active substances, the prevention and control of contaminating organisms in large-scale cultures of this alga are likely to receive increasing attention. Our research provides empirical evidence and a source reference to facilitate the detection of contaminants and the sustainable cultivation of *P. tricornutum*.

### Conclusions and significance.

A new heterolobosean amoeba, causing devastating losses in commercial *Phaeodactylum* cultures by predation, is described. Based on microscopic and ultrastructural features, molecular phylogenetic analysis, and the presence of two distinctive regions in the SSU rRNA gene, we establish a new species, *E. perlucida*. Considering its high grazing ability, wide food spectrum, capacity to form large populations in a short period of time, and capacity to form resistant cysts, *E. perlucida* has the potential to cause severe problems in pilot-scale microalgal/cyanobacterial cultures. To avoid losses in the yield or quality of the algal crop, further research should concentrate on developing efficient control and monitoring methods specific to *E. perlucida*.

## MATERIALS AND METHODS

### Algal strain and mass cultivation conditions.

The marine diatom *P. tricornutum* (strain UTEX640) was obtained from the Culture Collection of Algae at The University of Texas in Austin, TX, USA. *P. tricornutum* was maintained in F/2 medium (48) with a reduced salinity of 20‰. The media for pilot-scale photobioreactors were prepared using nonsterile tap water. Cultivation of *P. tricornutum* was carried out at the R&D facilities of the State Development & Investment Corporation Microalgae Biotechnology Center, Hebei, China (N 39°57′21.97″, E 116°51′35.95″). Light intensity and temperature changed with local weather conditions during the experimental period.

### Assessing the harmfulness of the amoeba on *P. tricornutum* mass culture in outdoor raceway ponds.

Investigations into the biological contamination of mass cultures of *Phaeodactylum* were conducted from 2018 to 2020. During the cultivation period, some protozoan predators contaminated the *Phaeodactylum* culture system (Table 1). This contamination always included an unknown amoeba predator, which occurred in different culture systems, including 520-L indoor semiclosed tubular photobioreactors, 150-L outdoor open raceway ponds, 1,300-L outdoor open raceway ponds, and 13,000-L outdoor open raceway ponds (Table 1). More than 70% of crashes of *P. tricornutum* cultures were caused by contamination with this amoeba.

To accurately evaluate the impact of the unknown amoeba on the biomass productivity of *P. tricornutum*, we studied two 13,000-L outdoor raceway ponds: one was contaminated by amoebae and was considered the experimental pond; the other was not contaminated by amoebae, or the concentration of amoebae was too low to be detected, and this was considered the control. We monitored the dry weight of *P. tricornutum* and the abundance of predatory amoebae every day during cultivation. In both ponds, the initial dry weight of *P. tricornutum* was adjusted to approximately 0.1 ± 0.01 g L^−1^ (approximately 3.5 × 10^6^ cells mL^−1^). The experiment lasted for 4 days. Light intensity and temperature changed with local weather conditions during the experimental period (Fig. S1). The cell dry weight (DW) of microalgal cultures was measured as described by Zhu and Lee (49). Samples (20 mL) were collected each day and filtered onto preweighed 0.45-μm GF/C superfine fiber membranes (1.2-μm pore size), with vacuum pressure differentials maintained at 35 to 55 mm Hg. The membrane was washed with 20 mL 0.5 M NH_4_HCO_3_ to remove nonbiological adhering materials such as mineral precipitates and was then dried at 105°C until a constant weight was obtained (about 24 h), cooled to room temperature in a vacuum desiccator, and weighed. For enumeration of amoebae, samples (1 mL) were stained with Lugol’s iodine (1% final concentration) (50). The cells (trophozoites and cysts of amoebae) were counted using 100-μL phytoplankton counting chambers (CC-F, China) under a phase-contrast microscope (BX53; Olympus, Japan) at ×400 magnification. Each sample was counted three times, and the mean value was used as the measure of abundance.

### Isolation and maintenance of the predatory amoeba.

Samples were collected from contaminated 13,000-L open raceway ponds to isolate the amoeba. Based on microscopic observations, single trophozoite amoebae were transferred into 12-well microtiter plates (catalog no. 40124; Beaverbio, China) containing *P. tricornutum* in F/2 medium to establish cocultures of amoebae and prey. These cocultures were maintained at 25°C under dim artificial light (a photon fluence rate of 5 to 20 μmol m^−2^ s^−1^) in a 12-h–12-h light-dark cycle. For long-term maintenance, 100 μL supernatant of a growing culture was transferred to algal material suspended in F/2 medium every 2 to 3 weeks. The amoebae and algal cultures are available from the Freshwater Algae Culture Collection at the Institute of Hydrobiology, Wuhan, China (http://algae.ihb.ac.cn/English/).

### Light-microscopic observation.

The morphological characters, life cycle, and feeding behavior of the amoeba, as well as the contamination process (described below), were observed with a Zeiss Axio Observer 3 inverted microscope (Zeiss, Oberkochen, Germany). In order to determine whether the amoeba has a flagellate stage, we set up experiments using various salinity gradients (3‰, 15‰, 30‰, 50‰, 75‰, 100‰, 150‰, 200‰, 250‰, and 300‰) and temperature gradients (5°C, 15°C, 25°C, 35°C, 45°C, and 55°C) to observe the cell morphology of the amoeba, as suggested by the previous studies (23, 24, 51, 52). Different salinities were achieved by increasing the concentration of sea salt based on the formulation of the F/2 medium. The medium used in the temperature gradient experiment was F/2 medium (salinity, 20‰). Well-growing cultures of *P. tricornutum* and amoebae were suspended in medium and distributed into 24-well microtiter plates (working fluid volume of 1 mL per well). All treatments were performed in triplicate. Experiments were carried out in a dark environment. The cellular morphology of amoebae was examined every 12 h throughout the 30-day experiment. To avoid cyst formation, food (*P. tricornutum*) was added every 3 days, and the culture system was refreshed every 7 days. High-resolution imaging and filming of real-time videos were performed with a Zeiss Axio Imager A2. Both microscopes were equipped with high-resolution differential interference contrast (DIC) optics and digital cameras (Zeiss Axiocam 506 color camera and SCMOS-pco Edge 4.2 LT). Adobe Photoshop CS5 (Adobe Systems, Munich, Germany) was used to adjust the color balance and contrast of light micrographs. Videos were analyzed and processed with Adobe Premiere Pro CC.

### Transmission electron microscopy.

The ultrastructure and digestion process of the amoeba were examined by transmission electron microscopy. Starved trophozoites, after-feeding trophozoites, and cysts of the amoeba were used to observe ultrastructure. The amoeba’s digestion process was monitored using the following method: well-grown *Phaeodactylum* cells were added to a large amount of a culture solution containing starved amoebae, mixed, and sampled, and samples were taken every 3 h thereafter. All samples were harvested by centrifugation (3,000 × *g* for 5 min; Eppendorf MiniSpin). Cells were then fixed with 2.5% glutaraldehyde overnight at 4°C. Following washing in phosphate buffer (0.1 M, pH 7.4) three times, cell samples were postfixed with 1% OsO_4_ for 1 to 2 h at room temperature. Subsequently, cells were quickly washed with phosphate buffer three times and dehydrated with a graded series of ethanol-water mixtures (50% ethanol, 70% ethanol, 90% ethanol, 100% ethanol, 100% ethanol), followed by a graded series of ethanol-acetone mixtures (25% acetone, 50% acetone, 75% acetone, 100% acetone, 100% acetone). Cells were then infiltrated with Spurr’s 812 epoxy resin, embedded, and cured in Spurr’s epoxy resin for 48 h at 60°C. The resulting samples (representing the hapantotype material for the new species) were sectioned (73 nm) using a Leica Ultracut-R microtome and stained with 2% uranyl acetate and Sato’s lead citrate (53). Sections were examined with a Hitachi HT-7700 (Japan) transmission electron microscope.

### DNA extraction, amplification, and phylogenetic analysis.

Starved individual trophozoites and cysts were isolated in a 0.2-mL PCR tube with 10 μL of Milli-Q water (one amoeba cell per tube, 60 tubes in total) and flash-frozen in liquid nitrogen. They were then subjected to single-cell PCR amplification. The SSU rRNA gene sequences were amplified using a combination of eukaryote primers (EukA, 5′-AACCTGGTTGATCCTGCCAGT-3′; EukB, 5′-TGATCCTTCTGCAGGTTCACCTAC-3′) (54). The samples were in a volume of 20 μL, containing 10 μL 2× GoTaq green master mix (Promega Corporation, USA), 0.5 μL of each primer (10 μM), 2 μL template DNA, 1 μL dimethyl sulfoxide, and 6 μL double-distilled water (ddH_2_O). The cycling conditions were as follows: an initial denaturing step at 94°C for 5 min, followed by 35 cycles of 30 s at 94°C, 1 min of annealing at 55°C, and extension at 72°C for 2 min, with a final extension step at 72°C for 10 min. Amplicons were approximately 2,000 bp for the SSU rRNA gene. They were checked on an agarose gel and purified with a gel extraction kit (no. 28704; Qiagen, Germany), ligated into a pGEM-T vector system (Promega, USA), and then transformed into competent cells. Ten positive clones were sent to be sequenced by a sequencing company (Tianyi Huiyuan, Wuhan, China). The inserted fragments were sequenced using M13 forward and M13 reverse universal primers.

For the phylogenetic analysis, the SSU rRNA gene sequences from 62 representative heterolobosean species were retrieved from the National Center for Biotechnology Information (NCBI) database (https://www.ncbi.nlm.nih.gov/). Multiple alignments of sequences were conducted using ClustalX 1.81 (55) and then manually arranged with Seaview (56). The alignment analyses were performed with DNAMAN (version 5.1) to determine any unique nucleotide regions for the amoeba, and the SSU rRNA secondary structure of Tetrahymena canadensis (accession number MW694332 [http://bioinformatics.psb.ugent.be/webtools/rRNA/secmodel/Tcan_SSU.html]) was used as a reference. After nonalignable sites were manually excluded, a data set with 1,689 sites was analyzed for the SSU rRNA gene sequences. Data sets were subjected to maximum-likelihood (ML) and Bayesian inference (BI) analyses under the GTR+Γ+I model (general time-reversible model with correction for invariant sites) (Modeltest 3.7) (57), using PhyML 3.0 (http://atgc.lirmm.fr/phyml/) and MrBayes 3.0b (58), respectively. For ML analyses, 100 independent tree searches and 1,000 bootstrap repetitions were performed. For the Bayesian analysis, the chain length was 5,000,000 generations, with trees sampled every 1,000 generations, and 25% (1,250,000 generations) were discarded as burn-in. The resulting phylogenetic tree, based on the best ML topology, contained support values from bootstrapping (ML) and posterior probabilities (BI) at the branches.

### Ability of the amoeba to feed on other microalgae and cyanobacteria.

The capacity of the amoeba to feed on diverse prey was qualitatively assessed using 28 strains of eukaryotic microalgae and eight cyanobacteria (Table 2). Eukaryotic microalgal strains included 13 chlorophytes, four chrysophytes, three euglenids, and four diatoms. All species were obtained from the Freshwater Algae Culture Collection (FACHB) and the Center for Microalgal Biotechnology and Biofuels at the Institute of Hydrobiology, Chinese Academy of Sciences, Wuhan, China. Well-growing cultures of microalgae and cyanobacteria were suspended in sterile F/2 medium and distributed into 12-well microtiter plates (approximately 3 mL per well). Starved amoebae were added, with the initial cell concentration of prey and starved amoebae being set at approximately 10^6^ cells mL^−1^ and 3 × 10^3^ cells mL^−1^, respectively, in each case. A positive control was established to assess the impact of the amoeba on *P. tricornutum* (UTEX640). For each algal strain, tests were conducted in triplicate, and the triplicates were independent biological replicates. Experiments were carried out at 25°C in a dark environment. The resulting cultures were monitored every day for a week to determine the following, as per More et al. (59): (i) whether amoebae were feeding, (ii) whether amoeboid populations were growing (assessed qualitatively), and (iii) whether the growth of amoebae was persistent (i.e., amoebae did not die before the food source was exhausted). Feeding was identified based on ingested prey cells or photosynthetic pigment material in trophozoites. The results were summarized in three categories: most suitable/rapid growth (++), suitable/limited growth (+), and not suitable/no growth (−).

In order to explore quantitatively the ability of the amoeba to graze on prey of different sizes, eight microalga/cyanobacterium strains (indicated in Table 2) that are nonmotile and unicellular and could be ingested well by amoebae were further selected as prey to assess the ingestion rate and growth rate of the amoeba. One of the strains was *P. tricornutum* UTEX640, which acted as a positive control. The remaining seven microalga/cyanobacterium strains included two chlorophytes (*Chlorella sorokiniana* CGMCC11801 and *Nannochloropsis oceanica* IMET1), one diatom (*Chaetoceros* sp. strain HR-CH301), and four cyanobacteria (Microcystis aeruginosa FACHB-905, *M. flos-aquae* FACHB-1028, *Synechocystis* sp. strain FACHB-898, and *Synechococcus* sp. strain FACHB-805). Cell diameters of the prey were measured using Zeiss microscope software, and the average diameter was calculated from 50 cells. For each strain, a suspension of the alga in sterile F/2 medium was added to 6-well microtiter plates (catalog no. 40106; Beaverbio, China; working fluid volume of 5 mL per well), with three control groups (containing only prey cells) and three experimental groups (containing both prey cells and amoebae) per strain. The initial cell concentration of algae in the control groups and experimental groups was always 2.0 (± 0.5) × 10^6^ cells mL^−1^, and the initial cell concentration of the amoeba in the experimental groups was always 1.5 (± 0.2) × 10^4^ cells mL^−1^. To eliminate the effect of photoautotrophic growth of algae on the analysis of ingestion rates, all cultures were cultivated in the dark. After cultivation/cocultivation for 36 h, cell concentrations of prey and the amoeba in all groups were counted as described in “Assessing the harmfulness of the amoeba on *P. tricornutum* mass culture in outdoor raceway ponds.”

The ingestion rate (*I*) of the amoeba on the algae and the growth rate (*G*) of the amoeba were calculated as described by Heinbokel (60): *I* = (*g* × *P*)/*D* and *G* = [ln(*D_t_*_1_) − ln(*D_t_*_0_)]/*t*, where *g* (specific grazing rate) is μ*_n_* − μ*_w_*. μ is [ln(*P_t_*_1_) – ln(*P_t_*_0_)]/*t*, in which μ*_n_* and μ*_w_* are the difference between net prey growth rate without and with grazers; *P_t_*_0_ and *P_t_*_1_ are the initial and final algal concentrations (in cells per milliliter), respectively; *P* (mean prey concentration) is (*P_t_*_1_ − *P_t_*_0_)/[ln(*P_t_*_1_) – ln(*P_t_*_0_)]; and *D* (mean predator concentration) is (*D_t_*_1_ − *D_t_*_0_)/[ln(*D_t_*_1_) – ln(*D_t_*_0_)], in which *D_t_*_0_ and *D_t_*_1_ are the initial and final *E. perlucida* concentrations (in cells per milliliter), respectively. *t* represents the time interval during which amoebae were in exponential growth phase. Here, the ingestion rate was used to evaluate the reduction in prey cells by grazing of per amoeba per hour, and growth rate represents exponential increase at a constant rate throughout time interval (60). Variation analysis of ingestion rate and growth rate were carried out by one-way analysis of variance (ANOVA) and Tukey’s test. The feeding rate and growth rate of amoebae were fitted with the average diameter of prey cells, respectively, and the fitting equation was obtained using SPSS 26.0.

### Nomenclatural acts.

This published work and the nomenclatural acts have been registered in ZooBank (http://zoobank.org/), the official online registration system for the ICZN. The ZooBank Life Science identifiers (LSIDs) can be resolved and the associated information viewed through any standard web browser by appending the LSID to the prefix http://zoobank.org/. The LSID for this publication is as follows: urn:lsid:zoobank.org:pub: 8FE9E9FE-43F2-470C-B666-4E47BDDB070A.

### Data availability.

The sequence determined here was deposited in GenBank and is available under the accession number OM654558.
