# Supplementary material for: A New Algivorous Heterolobosean Amoeba, Euplaesiobystra perlucida sp. nov. (Tetramitia, Discoba), Isolated from Pilot-Scale Cultures of Phaeodactylum tricornutum
Source: Microbiol Spectr. 2023 Jun 28;11(4):e00817-23. doi: 10.1128/spectrum.00817-23 (PMC10434026; doi:10.1128/spectrum.00817-23)
Supplement: Supplemental file 1 — Supplemental material. Download spectrum.00817-23-s0001.pdf, PDF file, 0.3 MB [file spectrum.00817-23-s0001.pdf]

Table. S1. Comparing the morphology of *Euplaesiobystra perlucida* sp. nov. with other species in the *Euplaesiobystra* genus

**Species**	**Adaptive type**	**Amoeba Length (μm)**	**Amoeba Width (μm)**	**Limax morphology**	**Uroidal filaments**	**Number of nuclei**	**Cyst characteristics**	**Plugged pore**	**Prey**	**Salinity at the isolation site**	**Referrences**
Euplaesiobystra perlucida	Amoeba; cyst	Average: 43.5 11.1–75.8	Average: 25.2 8.9–44.6	+	+	1-2	d=10.4–19.8 µm, average: 16.6µm; Young cyst: round, single cystwall; Mature cyst: round or irregular, double cystwall	1	Various eukaryotic algae and cyanobacteria	20-30‰	This study
Euplaesiobystra dzianiensis	Amoeba; cyst	Average: 22 17–33	Average: 13 8–20	+	+	1	d=8.6–10.4µm, average: 9.5µm; spherical or crescent; wall with distinct ectocyst and endocyst	1-2	*Arthrospira fusiformis*	40-60‰	1
Euplaesiobystra salpumilio	Amoeba; Flagellate; cyst	Average: 17.6 11.5–23.2	Average: 9.2 5.1–14.5	+	+	1	d=8.3–19.3 µm, average: 12.5µm; Spherical; thick endocyst and thin ectocyst; with a smooth outline of ectocyst	2-4	prokaryotes	134‰	2
Euplaesiobystra hypersalinica （*Plaesiobystra hypersalinica*）	Amoeba; Flagellate; cyst	Average: 30.5 19–41	Average: 12.0 9–16	+	+	1	d=14–20 µm, average: 16.8µm Approximately spherical; thick endocyst and thinner ectocyst, no cyst wall;	2-4	Prokaryotes, larger particles	293-300‰	3

NA: not available, +: presence.

References:

1. Aucher W, Delafont V, Ponlaitiac E, Alafaci A, Agogué H, Leboulanger C, Bouvy M, Héchard Y. 2020. Morphology and ecology of two new amoebae, isolated from a thalassohaline lake, Dziani Dzaha. Protist 171: 125770.

2. Lee HB, Jeong DH, Park JS. 2022. Accumulation patterns of intracellular salts in a new halophilic amoeboflagellate, *Euplaesiobystra salpumilio* sp. nov., (Heterolobosea; Discoba) under hypersaline conditions. Front Microbiol 13: 960621.

3. Park JS, Simpson AGB, Brown S, Cho BC. 2009. Ultrastructure and molecular phylogeny of two heterolobosean amoebae, *Euplaesiobystra hypersalinica* gen. et sp. nov. and *Tulamoeba peronaphora* gen. et sp. nov., isolated from an extremely hypersaline habitat. Protist 160: 265–283.

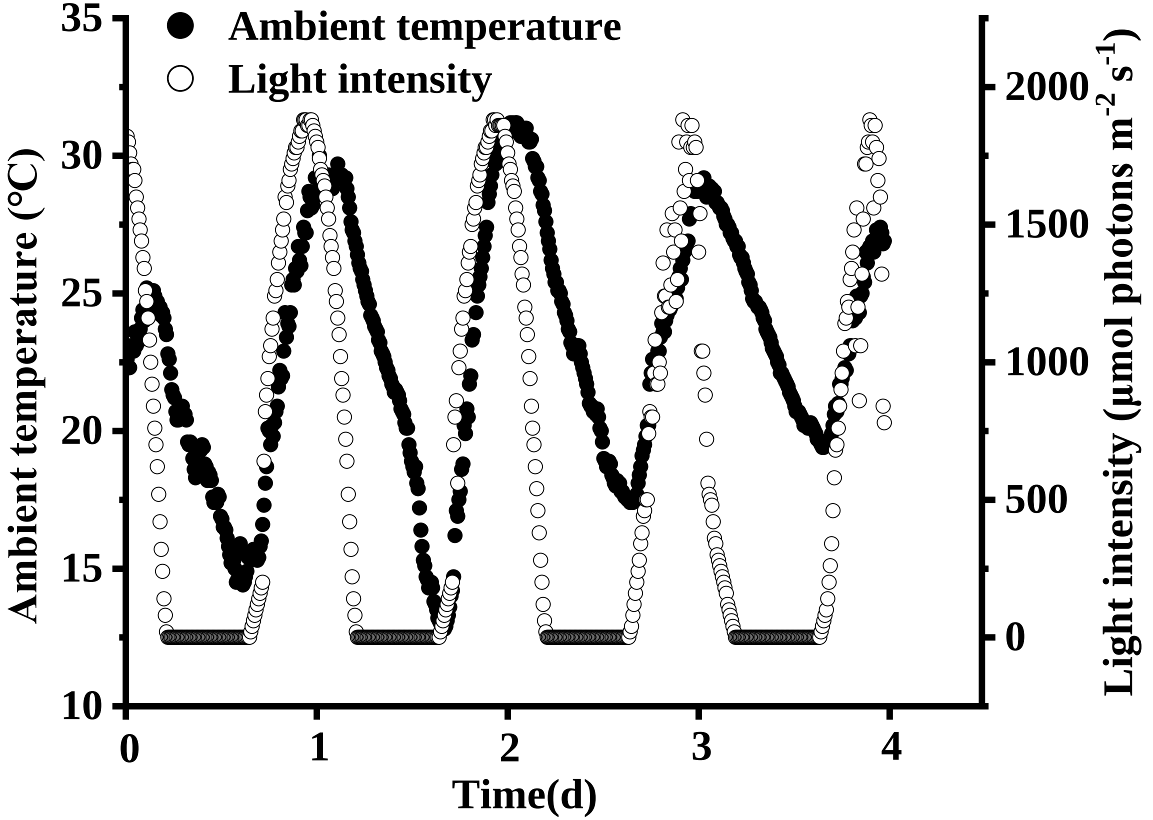

Fig. S1. Light intensity and ambient temperature during the outdoor (13,000-L open raceway ponds) experimental period at the R&D facilities of the State Development & Investment Corporation Microalgae Biotechnology Center, Hebei, China (N 39°57′21.97″, E 116°51′35.95″), from 30 April to 4 May 2019.

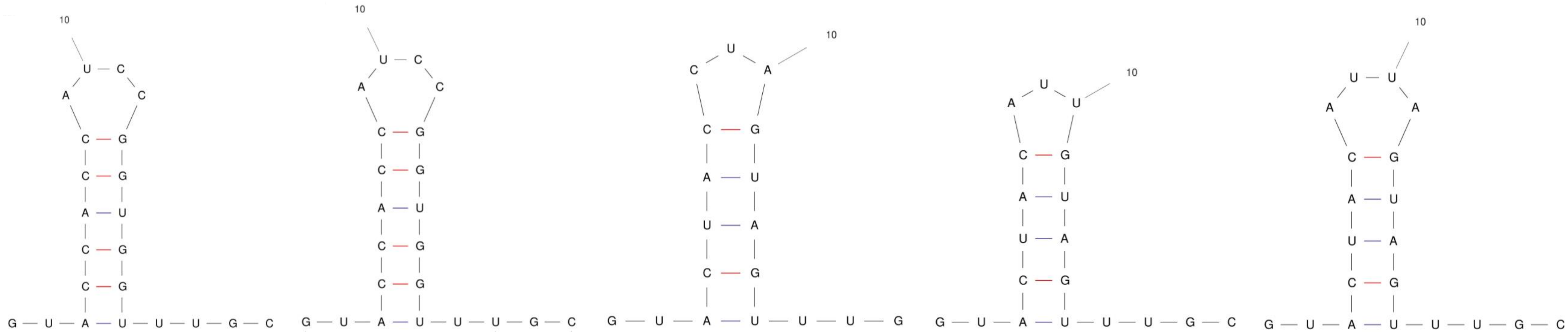

Uncultured heterolobosean H3 clone 4/8 KT210042

***Euplaesiobystra hypersalinica* FJ222604/Plaesiobystra hypersalinica AF011459**

***Euplaesiobystra dzianiensis* MN969059**

***Euplaesiobystra salpumillio strain ES010* ON561780**

***Euplaesiobystra perspicuous* OM654558**

Fig. S2. Putative secondary structures of the helix 17_1 in the SSU rRNA gene of the *Euplaesiobystra* group. Note that all heterolobosean species except *Pharyngomonas* strains has the helix 17_1 feature. Secondary structures were reconstructed using the UNAFold web server (http://www.unafold.org/).
